# Is leisure sedentary time associated with mental health issues? Evidence from China Health and Nutrition Survey

**DOI:** 10.3389/fpubh.2025.1517830

**Published:** 2025-02-06

**Authors:** Hao Li, Weihong Zeng

**Affiliations:** ^1^Jinhe Center for Economic Research, Xi'an Jiaotong University, Xi'an, Shaanxi, China; ^2^Center for Aging and Health Research, Xi'an Jiaotong University, Xi'an, Shaanxi, China

**Keywords:** risky health behavior, leisure sedentary time, mental health, health-promoting behavior, China

## Abstract

**Background:**

The relationship between leisure sedentary behaviors and mental health remains inconclusive. This study aims to provide evidence on leisure sedentary behaviors on mental health using longitudinal data, along with its moderating and mediating roles.

**Methods:**

We utilized data from four waves (2006–2015) of the China Health and Nutrition Survey (CHNS), focusing on adults who completed their education, with a total sample of 23,693 observations. Leisure sedentary time, the independent variable, was measured based on self-reported data, while mental health issues were the dependent variables based on the Simplified Symptom Self-Rating Scale (SCL) and the Perceived Stress Scale (PSS-14). The primary analysis employed ordinary least squares (OLS) regression. Potential endogeneity was addressed by instrumental variable (IV) method via two-stage least squares (2SLS) regression and a continuous difference-in-differences (DID) design.

**Results:**

Our findings indicate that high levels of sedentary time are associated adversely with mental health issues. Moderating roles show that improving health literacy and reducing sedentary time were effective in alleviating this adverse impact. Among older adults, social engagement and support from female caregivers showed greater potential to reduce the detrimental mental health impact of leisure sedentary time. Furthermore, obesity serve as its mediating role.

**Conclusion:**

This study highlights the potential adverse impact of increased leisure sedentary time on mental health among Chinese population. These results provide a foundation for public health initiatives aimed at addressing the rising prevalence of sedentary behavior and its association with mental health issues.

## Introduction

The rapid development of urbanization and technological advancements has led to a significant increase in sedentary behavior. Current estimates suggest that a large proportion of the adult population spends excessive amounts of time being sedentary (1). For example, a European survey reported that over 18% of adults sit for more than 7.5 h per day, with the figure ranging from 9% in Spain to 32% in the Netherlands (2). Similarly, Chinese adults spend an average of 8.5 h per day engaged in sedentary activities (3). As a result, the phrase “sitting is the new smoking” has been popularized in the media to highlight the growing epidemic across many nations (4). The relationship between sedentary behavior and physical health is well-established as the lack of utilization of body systems during sedentary time arguably leads to metabolic, hormonal, and muscular imbalances, which may attenuate the anti-inflammatory effects triggered by human musculature and promote systemic dysfunction (5). However, studies examining the relationship between sedentary behavior and mental health have produced inconsistent findings, despite extensive research on the topic (6, 7).

In addition to the prevalence of sedentary behaviors, there are several reasons to conduct a comprehensive investigation of how sedentary behavior affects mental health. First, inconsistent findings across studies hinder a clear understanding of this relationship. As noted by Higgins et al. (8) research on sedentary behavior is still in its early stages and requires broad inclusion criteria, especially longitudinal study (9). Second, according to the Report on the Development of Chinese National Mental Health (2019–2020), 12–20% of Chinese residents experience poor mental health (10). Unfortunately, a significant proportion of those with mental health issues do not receive the treatment they need (11), especially the impact of lifestyle habits on mental health is becoming increasingly important (12). Third, mental health strongly predicts self-rated health, surpassing physical health in its influence (13). Understanding mental health is, therefore, essential for improving overall wellbeing. Forth, leisure sedentary time is more random than occupational sedentary time, making it easier to detect statistically significant and reliable results.

Previous studies have revealed the relationship between lifestyle habits and mental health. A growing body of research highlights the influence of behaviors such as smoking, and alcohol consumption impact mental health (14). For example, studies point to the fact that smoking is a likely causal risk factor in the development of mental health issues (15). In addition, alcohol consumption is closely associated with the prevalence of depression (16). In particular, long-term heavy drinking can have serious emotional impacts and adverse consequences, including intentional self-harm and even suicide (17). Other factors, such as insufficient sleep, physical activity, and nutrition, also affect mental health (18). Insufficient sleep not only exacerbates mental health issues but may also interact with sedentary behavior, amplifying negative effects on inflammatory markers and psychophysiological health (19, 20). Additionally, research also suggests that physical activity and nutrition have a combined impact on mental health (21).

Regarding sedentary behavior, most studies suggest a correlation between prolonged sedentary time, particularly leisure-time screen use, and poorer mental health outcomes (22). For example, a large-scale study found that sedentary behavior increases the long-term risk of mental health disorders (23). Evidence links higher sedentary time with issues like depression and sadness, particularly when screen use is involved (24). Additionally, two Chinese studies using the CHNS data in 2015 and find increasing the time spent on leisure-time sedentary behavior is linked to lower perceived stress levels (25, 26). However, many of these studies are cross-sectional, limiting causal inferences (9). A longitudinal study following participants for seven years found that increased TV viewing heightened the odds of developing depressive symptoms, particularly among young men (27).

Contrary to this majority, some studies report no association between sedentary behavior and mental health, especially when accelerometer-based measurements are used (28, 29). Additionally, certain studies have found that TV viewing may have a positive role in mental wellbeing by fulfilling social, emotional, and informational needs, particularly during crises such as the COVID-19 pandemic (30). Despite these inconsistencies, the majority of evidence suggests that prolonged sedentary time is associated with an increased risk of mental health issues. Therefore, we propose the following hypothesis:

**Hypothesis 1:** Prolonged sedentary time is associated with a higher risk of mental health issues.

The relationship between sedentary behavior and mental health may be moderated by health literacy and efforts to reduce sedentary time. Health literacy, defined as the ability to obtain, process, and understand basic health information, significantly influences health outcomes. Higher health literacy can reduce psychological and social dysfunction and promote healthier lifestyles, including less sedentary behavior (31, 32). In addition, reducing or interrupting prolonged sitting is also a practical strategy for mitigating its negative effects. The World Health Organization (WHO) recommends minimizing sedentary time and incorporating regular interruptions (33), such as standing breaks during TV viewing or workplace strategies like walking meetings (34, 35). Based on the previous discussions, we propose the following hypothesis to examine moderating role:

**Hypothesis 2a:** Health literacy could diminish the negative effect of prolonged leisure sedentary time on mental health.
**Hypothesis 2b:** Reducing sedentary time could diminish the negative effect of prolonged leisure sedentary time on mental health.

The aging process brings physical and mental changes that profoundly affect quality of life. Older adults, who spend more time sitting and experience higher rates of mental health disorders, are particularly vulnerable (36, 37). In Chinese culture, where family is central (38), family caring and interactions may help mitigate the adverse effects of sedentary behavior on mental health (39). First, caring for elderly family members has been an essential function of families for centuries and is a moral requirement for adult children in some societies where family-oriented norms and values prevail (40). Beyond spouses, children—typically daughters—are the most common informal caregivers (41) as societal expectations and gender norms (42, 43). For instance, Zhao et al. (44) found that families with daughters tend to provide higher-quality informal care than those with sons, particularly for older adults with greater care needs. Second, older adults with stronger social interactions have an increased likelihood of better health and higher increase in mental wellbeing than the socially disengaged (45). Social interactions might encourage people to adopt healthy behaviors, such as various physical activities, that benefit their mental health (46, 47), and social interactions might enhance the diffusion of health-related information by encouraging social interaction among neighbors (46, 48). Based on the previous discussions and analyses, we propose the following hypothesis to examine moderating role:

**Hypothesis 3a:** Female caregivers are more likely to diminish the negative effect of prolonged leisure sedentary time on mental health.
**Hypothesis 3b:** Social interaction could diminish the negative effect of prolonged leisure sedentary time on mental health.

After discussing the moderating roles between sedentary time and mental health, we turn our focus on mediating role. Sedentary behavior may indirectly affect mental health through obesity as sedentary activities are low-energy activity (49). Additionally, obesity, biologically, is associated with chronic low-grade inflammation in peripheral tissues (50), thereby leading to depression (51). Based on the previous discussions and analyses, we propose the following hypotheses:

**Hypothesis 4:** Obesity mediates the relationship between sedentary behavior and mental health.

In summary, this paper examines the relationship between leisure sedentary time and mental health using representative longitudinal data. In addition, to further understand this relationship, we also explore both moderating and mediating effects.

## Data and empirical strategy

### Data

The China Health and Nutrition Survey (CHNS) is an ongoing large-scale study aimed at determining how China's social and economic development affects the health and nutritional status of the country's population. It is conducted by the Carolina Population Center at the University of North Carolina in Chapel Hill and the National Institute for Nutrition and Health (formerly the National Institute of Nutrition and Food Safety) at the Chinese Center for Disease Control and Prevention. A multistage, random cluster process was used to draw a sample of ~7,200 households comprising more than 30,000 individuals across 15 provinces and municipal cities. The CHNS data-collection rounds were completed in 1989, 1991, 1993, 1997, 2000, 2004, 2006, 2009, 2011, and 2015. Our analysis draws on four waves-2006, 2009, 2011, and 2015—during which data on mental health issues and sedentary behavior were available. We focus on adults who have completed their formal education, resulting in a total of 23,693 observations.

### Variables

#### Mental health issues

The CHNS collected two mental health scales across different years: the Simplified Symptom Self-Rating Scale (SCL) in 2006, 2009, 2011, and 2015 for individuals aged 45 and older, and the Perceived Stress Scale (PSS-14) in 2015 for all adults. To create comprehensive mental health measures, we combined scores from both the SCL and PSS-14 and then standardized them to a mean of zero and a standard deviation of one. Those two metrics were used as dependent variables in our main regressions.

Psychological wellbeing (PWB) is an essential and integral component of health, which is related to cognitive function, social relationships and health status and is a combination of feeling good and effective functioning (52). The PWB was measured by asking participants to answer three questions: “I have as much energy as I had in last year”; “I am as happy now as when I was younger”; “As I get older, things are better than I thought they would be.” Responses were scored on a five-point scale (1–5). To align with the trend of PSS-14, reverse scoring was applied, where higher scores indicate more frequent depressive symptoms and poorer mental health.

Can this newly constructed indicator reflect an individual's mental health relatively accurately? From the content point of view, the first question reflects the individual's health status and is a simplified index of somatization in SCL-90 (mainly reflecting subjective physical discomfort). The second question reflects the individual's psychological state and is a simplified measure of depression, anxiety, hostility, and terror in SCL-90. The third question is the individual's perceptions and attitudes toward the world around them, which is a broader indicator. Therefore, these questions are a great simplification of SCL-90 (53). The SCL has been widely used in previous studies [e.g., (53–55)].

Perceived stress is another important mental health indicator, manifesting as tension and discomfort in response to stressful situations or unfavorable life conditions (56). Prior studies have pointed out that perceived stress is positively associated with depression (57) and anxiety (58). In the 2015 wave, the PSS-14 was incorporated into the project for the first time (26). The PSS-14 is a validated questionnaire developed by Cohen et al. (59), and its Chinese version has been validated (60). It aims to measure the degree to which situations in one's life are appraised as stressful, and the items are designed to measure the extent to which one's life is perceived as unpredictable, uncontrollable, and overloading (59). Responses were scored on a 5-point Likert scale from 1 (“never”) to 5 (“very often”). Scores were calculated by reverse-scoring the positively stated items (4, 5, 6, 7, 9, 10, and 13) and summing the 14 items for a total score. Higher scores indicate greater perceived stress.

#### Leisure sedentary time

Sedentary behavior can be measured using subjective or objective methods. In this study, we use self-reported leisure sedentary time as the independent variable due to data constraints. Over recent decades, the ownership of color televisions and access to cable networks among Chinese households have grown significantly (61). During the survey period, more than 90% of Chinese families owned a television (62), making TV-based leisure sedentary time a valid measure.

The CHNS collects data on leisure sedentary time using a single question. Adult participants were asked, “Do you participate in these sedentary activities?” The listed activities included watching TV, watching videos, and playing video games. Participants then provided details on the time spent on these activities, answering two follow-up questions: “How much time do you spend during Monday to Friday?” and “How much time do you spend during Saturday to Sunday?” From these responses, we calculated the total leisure sedentary time. Studies commonly use non-exercise sitting behaviors, especially screen-based activities, to measure sedentary behavior (63). These measures typically show acceptable reliability and validity (64) and have been widely used in previous studies [e.g., (62, 65)].

In our analysis, we computed the independent variable using Equation 1 and applied winsorization at the 1st and 99th percentiles of the sample distributions. The equation is as follows:


(1) $$\begin{array}{l} x_{it}={x\left(weekday\right)}_{it}*5/7+{x\left(weekend\right)}_{it}*2/7 \end{array}$$

#### Control variables

Referring to prior population-based studies (66, 67), we control for a comprehensive set of individual-, household-, and macro-level characteristics. First, we include individual and household factors such as marital status, sleep duration, physical activity, smoking status, drinking status, body mass index (BMI), self-reported health status, chronic status, and log-transformed annual household income. Additionally, we account for occupational sedentary time, which significantly affects mental health (68). To address multicollinearity issues, we standardize occupational sedentary time to a mean of zero and a standard deviation of one, given its inverse relationship with leisure sedentary time. Dietary factors are also included, as evidence suggests certain dietary patterns positively impact mental health. A balanced diet, rich in vegetables, fruits, whole grains, seafood, nuts, and moderate amounts of low-fat dairy, red meat, and healthy fats, is associated with better mental health outcomes (69). Moreover, dietary quality has been linked to the presence or progression of depressive symptoms (70). To capture dietary influences, we include variables for dietary preferences, a dietary knowledge index, and key nutritional elements. Dietary preferences are measured using four variables: preference for fast food, salty food, vegetables, and sugary foods, each scored from 1 to 5. Higher scores indicate a stronger preference. The dietary knowledge index, constructed from a rich set of dietary knowledge variables, reflects better dietary understanding, with higher scores indicating greater knowledge^1^. We also control for average daily intake of dietary fat and protein (in grams), where healthier dietary preferences and a higher dietary knowledge index suggest better dietary patterns and quality. Furthermore, to mitigate omitted variable bias related to genetic predisposition, we control for individual disease diagnoses, including mental health and genetic disorders. At the macro level, we include Urbanization, Economic, Health, and Housing Scores, where higher scores represent better development in these domains. Additionally, longitudinal studies are better to disentangle a temporal relationship between sedentary time and mental health issues (71). Thus, we control for year fixed effect. To address the existence of spatial heterogeneity in mental health issues, we also control for fixed effect at individual-level, household-level, and community-level. Age effects are absorbed by these fixed effects.

Table 1 summarizes the descriptive statistics for the sample. The average aggregated mental health score is 22, with a range from 1 to 78. Participants spend an average of 143 min per day in leisure sedentary activities. The average sleep duration is 7.81 h per day, and the mean BMI is 24, ranging from 13 to 32. Additionally, the proportions of individuals who exercise, smoke, drink alcohol, have chronic diseases, or are diagnosed with mental health or genetic disorders are relatively low, averaging 0.12, 0.31, 0.31, 0.30, and 0.01, respectively. Most participants are married, and the average annual household income is 51,660 yuan. Regarding self-reported health status, the average self-reported health score is 2, suggesting that most individuals perceive their health as good. Regarding dietary patterns and quality, most participants report disliking fast food, salty foods, and desserts, while most favor vegetables. The average dietary knowledge index is 22. Average daily intake of dietary fat and protein is 70 g and 64 g, respectively. For macro-level variables, the average Urbanization, Economic, Health, and Housing Scores are 71, 8, 9, and 6, respectively.

**Table 1:** Summary statistics of key variables.

	**Definition (%)**	**Mean (SD)**
**Outcomes**		
Mental health 1	Aggregated mental health scores	22 (17)
Mental health 2	Standardized mental health scores	0 (1)
**Independent variable**		
Leisure sedentary time	The average leisure sedentary time (mins)	143 (96)
**Covariates**		
Marital status	0 = Otherwise (13); 1 = Married (87)	0.87 (0.34)
Sleep time	Sleep time (hours)	7.81 (1.23)
Physical activity	0 = Never exercise (89); 1 = Participate in one sport (9); 2 = Participate in two sports (1); 3 = Participate in three sports (1)	0.12 (0.38)
Smoke status	0 = No (69); 1 = Yes (31)	0.31 (0.46)
Alcohol status	0 = No (69); 1 = Yes (31)	0.31 (0.46)
BMI	Bady mass index	24 (3)
Self-reported health	1 = very good (19); 2 = good (46); 3 = fair (29); 4 = bad (5); 5 = very bad (1)	2 (0.82)
Chronic diseases	0 = No chronic diseases (78); 1 = Hypertension or diabetes (20); 2 = Hypertension and diabetes (2)	0.30 (1.02)
Disease diagnosis records	Whether the individual has a diagnosed mental health or a diagnosed genetic disease	0.01 (0.09)
Occupational ST	Standardized average weekly working time	0 (1)
Preference for fast food	1 = strongly dislike fast food (19); 2 = dislike fast food (68); 3 = neutral (9); 4 = like fast food (3); 5 = strongly like fast food (1)	1.97 (0.65)
Preference for salty food	1 = strongly dislike salty food (14); 2 = dislike salty food (67); 3 = neutral (13); 4 = like salty food (5); 5 = strongly like salty food (1)	2.11 (0.70)
Preference for vegetables	1 = strongly dislike eating vegetables (1); 2 = dislike eating vegetables (1); 3 = neutral (11); 4 = like eating vegetables (76); 5 = strongly like eating vegetables (7)	3.91 (0.53)
Preference for dessert	1 = strongly dislike eating dessert (7); 2 = dislike eating dessert (54); 3 = neutral (25); 4 = like eating dessert (13); 5 = strongly like eating dessert (1)	2.45 (0.83)
Dietary knowledge index	Aggregated dietary knowledge scores	22 (3)
Average fat intake	Average fat intake (g/d)	70 (30)
Average protein intake	Average protein intake (g/d)	64 (19)
Family income	Annual household income (RMB yuan)	51,660 (48,513)
Urbanization	Urbanization index	71 (19)
House scores	House scores	8 (2)
Economic scores	Economic scores	9 (3)
Health scores	Health scores	6 (3)

### Baseline model

We choose the ordinary least squares (OLS) method to test the relationship between leisure sedentary time and mental health issue in Hypothesis 1. The equation for the method is as follows (see Equation 2):


(2) $$\begin{array}{l} Y_{it}={\beta_{0}+\beta }_{1}ST_{it}+\beta_{2}X_{it}+\delta_{i}+\delta_{f}+\delta_{commid}+\tau_{t}+\varepsilon_{it} \end{array}$$

Where *Y*_*it*_ is dependent variable for individual *i* at wave *t*, and *ST*_*it*_ represents individual *i*'s leisure sedentary time at wave *t*. *X*_*it*_ is a set of control variables. δ_*i*_, δ_*f*_, and δ_*commid*_, represent the fixed effects at individual-level, household-level, and community-level, respectively. τ_*t*_ represents year-fixed effect. β_1_ is the main coefficient of interest, β_1_>0 indicates that heightened leisure sedentary time negatively affects mental health issues.

### Discussion of endogeneity

Prior studies suggest that inconsistent findings regarding the relationship between sedentary time and mental health may stem from confounding factors, causality issues, and measurement errors (72). This necessitates a comprehensive review of endogeneity concerns.

One potential source of endogeneity, in the context of our study, is omitted variable bias. There are several unobservable factors that are likely to be correlated with both leisure sedentary time and mental health issues and, thus, in a multivariate regression framework, it is difficult to rule out more than one omitted variable, making it impossible to clearly predict the direction of bias. For example, adverse childhood experiences are associated with both higher sedentary behaviors, particularly greater screen time, and worse health conditions (73, 74).

While the fixed effect model is generally known to be effective in addressing omitted variable bias, endogeneity may also emerge from potential measurement error and simultaneity bias. Regarding measurement error bias, previous literature has found that leisure sedentary time was collected through questionnaires, inherently resulting in an underestimation of leisure sedentary time (75). Therefore, $\hat{\beta_{1}}$ will underestimate β_1_ if β_1_ is positive. Concurrently, emerging evidence suggests bidirectional associations between sedentary behaviors and mental health issues (76). Individuals with mental health issues are generally less active, and sedentary behavior increases the risk of mental health issues. Thus, simultaneity bias should be considered.

Good instrumental variables must satisfy two assumptions. The first assumption is that they should be strongly correlated with the variable being instrumented. The second assumption is that the instrumental variables are orthogonal to the outcomes. To meet the above assumptions, this paper employs three instrumental variables.

First, building on Munasib and Bhattacharya (77), we adopt Lewbel's higher-moment approach (78). This method, initially designed for measurement error models, is widely applicable in contexts with general correlated-regressor error and multilevel structures (79). It constructs instrument using the third-order centered moment of leisure sedentary time. By design, this variable is strongly correlated with leisure sedentary time but unlikely to be correlated with mental health issues unless a non-linear relationship exists. This approach has been extensively validated in prior studies [e.g., (80, 81)].

Second, Duflo and Saez suggest using the average exogenous characteristics of group-level variables as instruments for endogenous independent variables (82). Therefore, inspired by the peer group effect theory (83), we employ the average leisure sedentary time in the household of individual *i* as second instrumental variable (group-level IV). The average leisure sedentary time in the household of individual *i* is strongly influence individual *i*'s leisure sedentary time under the family norms. However, this variable is unlikely to correlate with individual time-varying unobservable characteristics once fixed effects are accounted for (84).

The third instrument is the number of TV channels available to a household. This variable is exogenous, as the availability of television signals in China is determined by national and local broadcasting authorities. At the same time, it is associated with the amount of time spent watching television, making it a valid instrument for leisure sedentary time.

These IVs collectively address the potential endogeneity issues in the relationship between sedentary time and mental health outcomes.

### DID method

Despite we employ instrumental variable method to address endogenous concerns, the results are still affected by unobserved factors. Therefore, we draw from National Digital TV Development Plan as an exogenous variation to examine the effect of leisure sedentary time on mental health issues under the continuous difference-in-differences (DID) method. The logic of DID method is that the prevalence of high-definition digital television after policy greatly affects the screen time, thereby affecting mental health.

To meet the demand for high-definition television broadcasts of the 2008 Beijing Olympics, the State Administration of Radio, Film, and Television (SARFT) accelerated the promotion of high-definition digital television. High-definition TV programming and event broadcasts required compatible digital TV and set-top boxes, which significantly boosted demand for set-top boxes. As early as 2003, SARFT released the Digital TV Development Plan and accelerated its implementation in 2008. The plan mandated that by 2010, the digitalization of cable television networks should be largely completed. This target pushed local television stations to speed up the rollout of digital TV. Meanwhile, SARFT reinforced the requirement for the gradual shutdown of analog television signals in 2008, promoting the replacement of analog TV with digital TV. This policy further drove demand for set-top boxes since consumers needed them to receive digital signals. Financially, to accelerate the adoption of digital TV, some local governments introduced subsidy policies for set-top boxes in 2009. Consumers could receive price discounts when purchasing set-top boxes, which further boosted sales. Considering the timing of the plan, we choose 2009 as the cutoff. The regression framework of the continuous DID design is typical written as Equation 3 as follows.


(3) $$\begin{array}{l} Y_{it}={\beta_{0}+\beta }_{1}ST_{it}*D_{it}+\beta_{2}X_{it}+\delta_{i}+\delta_{f}+\delta_{commid}+\tau_{t}+\varepsilon_{it} \end{array}$$

Where the dummy variable *D*_*it*_ is equal to 0 for years from 2009 onwards and 1 otherwise, and other specifications are similar to Equation 2.

## Results

### Baseline results

To examine the relationship between leisure sedentary time and mental health issues, we estimate Equation 2, with results presented in Table 2. Column (1) uses aggregated mental health scores as the dependent variable, while column (2) uses standardized mental health scores. Both coefficients are positive and statistically significant. Specifically, we find that, on average, one additional minute of leisure sedentary time increases aggregated mental health scores by 0.0010 and standardized mental health scores by 0.0001. A back-of-the-envelope calculation indicates that one day of sedentary behavior increases aggregated mental health scores by 0.1430 and standardized scores by 0.0143. These findings suggest a consistent conclusion: increased leisure sedentary time is associated with mental health issues, supporting Hypothesis 1. However, these results should be interpreted cautiously, as the baseline estimation may be affected by endogeneity issues.

**Table 2:** Results of baseline regressions about the impact of leisure sedentary time on mental health issues (OLS).

	**Mental health 1**	**Mental health 2**
Sedentary	0.0010^*^	0.0001^*^
	(0.0006)	(0.0000)
**Individual characteristic**		
Marital status	−0.6798^**^	−0.0405^**^
	(0.3259)	(0.0194)
**Life habits**		
Sleep time	−0.0483	−0.0029
	(0.0437)	(0.0026)
Physical activity	−0.1026	−0.0061
	(0.1538)	(0.0092)
Smoke status	−0.4035^**^	−0.0240^**^
	(0.1987)	(0.0118)
Alcohol status	−0.1183	−0.0070
	(0.1650)	(0.0098)
**Physical index**		
BMI	−0.0347	−0.0021
	(0.0330)	(0.0020)
Self-reported health	0.8285^***^	0.0493^***^
	(0.1046)	(0.0062)
Chronic diseases	−0.2183	−0.0130
	(0.1501)	(0.0089)
Disease diagnosis records	1.4548^***^	0.0866^***^
	(0.5517)	(0.0328)
**Occupational characteristic**		
Occupational ST	−0.2378^***^	−0.0142^***^
	(0.0662)	(0.0039)
**Dietary characteristics**		
Preference for fast food	−0.0368	−0.0022
	(0.1086)	(0.0065)
Preference for salty food	0.0008	0.0000
	(0.0957)	(0.0057)
Preference for vegetables	−0.7660^***^	−0.0456^***^
	(0.1005)	(0.0060)
Preference for dessert	0.2564^***^	0.0153^***^
	(0.0694)	(0.0041)
Dietary knowledge scores	0.0055	0.0003
	(0.0186)	(0.0011)
Average fat intake	0.0023	0.0001
	(0.0022)	(0.0001)
Average protein intake	0.0006	0.0000
	(0.0035)	(0.0002)
**Household characteristic**		
Family income	−0.1725^***^	−0.0103^***^
	(0.0570)	(0.0034)
**Regional characteristic**		
Urbanization	−0.0172	−0.0010
	(0.0105)	(0.0006)
House scores	0.2289^***^	0.0136^***^
	(0.0749)	(0.0045)
Economic scores	−0.0464	−0.0028
	(0.0323)	(0.0019)
Health scores	−0.0802^***^	−0.0048^***^
	(0.0302)	(0.0018)
Fixed-effects	Yes	Yes
Constant	25.6389^***^	0.2274^***^
	(1.4275)	(0.0850)
Obs.	23,693	23,693
R^2^	0.960	0.960

Significance: ^*^, ^**^, and ^***^ denote significance at 10%, 5%, and 1% level, respectively.

Fixed-effects include individual-level, household-level, and community-level, and year dummy.

Our analysis also shows that marriage is strongly associated with better mental health. This aligns with existing evidence that marriage improves health through mechanisms such as social support, health behavior regulation, and access to resources that protect health (85). Similarly, physical health, higher family income, and better health scores are positively associated with improved mental health. In contrast, higher housing scores are negatively associated with mental health. One possible explanation is that rapidly rising housing prices in China during the survey period increased stress and mental health risks. Furthermore, individuals diagnosed with mental health issues or genetic disorders tend to report poorer mental health outcomes. Interestingly, we find that smoking is positively associated with mental health. While most studies highlight the adverse impact of smoking on mental health, while some smokers report that smoking helps improve mood, reduce stress, or act as a form of coping (86). We also observe that occupational sedentary time is positively linked to mental health. This may be explained by a shift in sedentary behavior patterns during the survey waves, where leisure sedentary time increased while occupational sedentary time decreased (87). Lastly, dietary characteristics are significantly associated with mental health. For instance, individuals who prefer vegetables tend to have better mental health, while those with a preference for sweets report poorer mental health outcomes.

### Instrumental variable results

To alleviate endogenous concerns, Table 3 shows the instrumental variable regression results. Column (3) shows the first-stage IV estimates, indicating a significant positive relationship between the instruments and the endogenous independent variable. The first-stage F-test yields an F-statistic of 1,541, which exceeds the threshold for weak instruments. Additionally, the *p*-value of the Kleibergen-Paap rk LM statistic is 0.000, indicating that the null hypothesis is rejected, and the instrumental variable passes the unrecognizable test. Concurrently, Sargan test *p*-values exceed 0.1, confirming the exogeneity of the instrumental variables. Hence, our instrumental variable selection is reasonable. Moving to column (1) and column (2), the result show that the regression coefficients are 0.0017 and 0.0001, respectively, and are significant at the 1% level, which are consistent with the baseline regression. Specially, the coefficients in column (1) and column (2) are larger than those in Table 2, suggesting a potential downward bias in the estimates when endogeneity is not addressed. To examine the valid of Lewbel's IV, Table 4 further reports the results of non-linear relationship between leisure sedentary time and mental health issues. The results show this non-linear relationship is unlikely to be hold, confirming the validity of Lewbel's IV.

**Table 3:** Endogenous test of the effect of leisure sedentary time on mental health issues (IV).

	**Mental health 1**	**Mental health 2**	**First stage**
Sedentary	0.0017^***^	0.0001^***^	
	(0.0007)	(0.0000)	
Lewbel's IV			0.0000^***^
			(0.0000)
Group-level IV			0.7334^***^
			(0.0068)
The numbers of TV channels			0.1132^*^
			(0.0657)
Covariates	Yes	Yes	Yes
Fixed-effects	Yes	Yes	Yes
Constant	25.5553^***^	0.2224^***^	5.7367
	(1.4276)	(0.0850)	(11.1466)
F-stat (First stage)			1,541
Kleibergen-Paap rk LM *P*-value			0.000
Sargan test *P*-value			0.127
Obs.	23,693	23,693	23,693
R^2^	0.960	0.960	0.926

Significance: ^*^, ^**^, and ^***^ denote significance at 10%, 5%, and 1% level, respectively.

All models include fixed effects and all covariates.

**Table 4:** The non-linear relationship between leisure sedentary time and mental health issues to examine the validity of Lewbel's IV.

	**Mental health 1**	**Mental health 2**
Sedentary	−0.0016	−0.0001
	(0.0017)	(0.0001)
Sedentary-squared	0.0000^*^	0.0000^*^
	(0.0000)	(0.0000)
Covariates	Yes	Yes
Fixed-effects	Yes	Yes
Constant	25.7981^***^	0.2369^***^
	(1.4305)	(0.0852)
Obs.	23,693	23,693
R^2^	0.960	0.960

Significance: ^*^, ^**^, and ^***^ denote significance at 10%, 5%, and 1% level, respectively.

All models include fixed effects and all covariates.

### DID method results

Table 5 further presents the results of DID method. The result of the first two columns show that heightened leisure sedentary time is linked with worse mental health issues. Similarity, the coefficients of the first two columns are larger than those in Table 2, suggesting a potential downward bias in the estimates when endogeneity is not addressed. A back-of-the-envelope calculation suggests that one-day sedentary behavior significantly increases in aggregated mental health scores by 0.315 on average, and standardized mental health scores by 0.0143. A basic premise for using the DID model to evaluate policy effects is that the development trends of the explained variables for the treatment and control groups should be consistent before policy implementation in the pilot. In other words, there should be no statistically significant difference between the treatment and control groups before policy implementation. Therefore, it is necessary to conduct a parallel trend test for the DID model. In this study, we use the period prior to policy implementation, specifically 2009, as the reference group. As shown in Column (3), the coefficients of the parallel trend are insignificant before the policy. In 2011, the first period after policy implementation, the coefficients of the parallel trend are insignificant, which may be attributed to policy lag. However, in the second period after policy implementation, namely in 2015, the coefficients of the parallel trend become statistically significant. It indicates that the DID model in this study passes the parallel trend test, thereby validating the regression results presented in Table 5 to some extent.

**Table 5:** Results of DID regressions about the impact of leisure sedentary time on mental health issues.

	**Mental health 1**	**Mental health 2**	**Parallel trends**
Sedentary	0.0022^***^	0.0001^***^	
	(0.0008)	(0.0000)	
2006			0.0002
			(0.0001)
2009			−0.0001
			(0.0001)
2011			0.0000
			(0.0001)
2015			0.0002^***^
			(0.0001)
Covariates	Yes	Yes	Yes
Fixed-effects	Yes	Yes	Yes
Constant	25.5424^***^	0.2217^***^	0.2248^***^
	(1.4273)	(0.0850)	(0.0850)
Obs.	23,693	23,693	23,693
R^2^	0.960	0.960	0.960

Significance: ^*^, ^**^, and ^***^ denote significance at 10%, 5%, and 1% level, respectively.

All models include fixed effects and all covariates.

### Moderating effect

The empirical results show that increased leisure sedentary time negatively impacts mental health. Therefore, mitigating this adverse effect is a critical goal for policy interventions and health-promoting behaviors. In this section, we examine moderating factors that may reduce the negative impact of leisure sedentary time on mental health.

First, we examine the moderating role of health literacy. In doing so, we use two measures: whether individuals are familiar with the Chinese Dietary Guidelines (CDG) and whether they have family members who are medical professionals. Familiarity with the CDG reflects a tendency to seek health-related information, while having medical professionals in the family indicates greater health literacy within the household. Based on that, we divide the sample into two groups: those unfamiliar with the CDG vs. those familiar, and households without medical professionals vs. those with medical professionals. Table 6 presents the results. For individuals unfamiliar with the CDG, the coefficient for leisure sedentary time is significantly positive at the 10% level. In contrast, for those familiar with the CDG, the coefficient is positive but not statistically significant at the 10% level. Similarly, households without medical professionals show a significantly positive coefficient at the 5% level, while households with medical professionals exhibit an insignificant coefficient, despite its larger magnitude. These findings indicate that health literacy moderates the relationship between leisure sedentary time and mental health issues, supporting Hypothesis 2a.

**Table 6:** Moderating effect—health literacy.

	**Not knowing CDG**	**Knowing CDG**	**Without medical workers**	**Having medical workers**
Sedentary	0.0014^*^	0.0013	0.0016^**^	0.0033
	(0.0007)	(0.0026)	(0.0007)	(0.0047)
Covariates	Yes	Yes	Yes	Yes
Fixed-effects	Yes	Yes	Yes	Yes
Constant	25.0142^***^	31.1075^***^	25.7426^***^	34.9139^***^
	(1.6229)	(6.2375)	(1.4546)	(11.6775)
Obs.	18,529	5,104	22,637	1,056
R^2^	0.963	0.982	0.959	0.986

Significance: ^*^, ^**^, and ^***^ denote significance at 10%, 5%, and 1% level, respectively.

The dependent variable is Mental health 1.

All models include fixed effects and all covariates.

Next, we examine whether reducing prolonged sedentary time improves mental health. We first divide the sample based on the number of accessible TV channels, assuming a correlation between leisure sedentary behavior and television availability. Using the National Digital TV Development Plan as a benchmark, we categorize samples into two groups based on the average number of TV channels before and after 2009 (23 vs. 31 channels). Second, leveraging the longitudinal nature of the CHNS data, we group individuals based on changes in sedentary time over survey years, distinguishing between increased and reduced sedentary time. Table 7 presents the regression results. In the first two columns, households with more TV channels show a stronger negative impact of sedentary time on mental health, reflected by larger coefficients and greater statistical significance. In the last two columns, reduced sedentary time is associated with larger coefficients, though not statistically significant, while increased sedentary time has significant negative effects with a greater magnitude than the baseline regression. These results support Hypothesis 2c.

**Table 7:** Moderating effect—changes in leisure sedentary time.

	**Fewer TV channels**	**More TV channels**	**Sedentary time decrease**	**Sedentary time increase**
Sedentary	0.0021^*^	0.0028^***^	0.0048	0.0032^***^
	(0.0012)	(0.0009)	(0.0038)	(0.0011)
Covariates	Yes	Yes	Yes	Yes
Fixed-effects	Yes	Yes	Yes	Yes
Constant	16.6528^***^	29.9125^***^	25.2591^***^	24.0061^***^
	(2.5457)	(2.0067)	(4.1960)	(2.5894)
Obs.	6,387	17,306	7,064	9,976
R^2^	0.969	0.962	0.984	0.976

Significance: ^*^, ^**^, and ^***^ denote significance at 10%, 5%, and 1% level, respectively.

The dependent variable is Mental health 1.

All models include fixed effects and all covariates.

Finally, we focus on older adults and examine the moderating roles of caregiver gender and social interaction. To investigate Hypothesis 3b, we use the gender composition of caregivers. Older adults are assumed to interact more with female caregivers when daughters constitute the majority of caregivers, as the CHNS does not explicitly ask about the primary caregiver's gender. We also examine the role of urban parks as facilitators of social interaction. Parks provide spaces for physical activity, relaxation, and community engagement, which enhance mental health and wellbeing (88). While beneficial for all age groups, parks hold particular significance for older adults (89). Using the question “Does your house have a park nearby?”, we classify older adults into two groups: those with nearby parks and those without. We hypothesize that proximity to parks promotes social activities, reduces sedentary time, and improves mental health. Table 8 reports the results. In households with predominantly female caregivers, older adults experience fewer mental health issues, as evidenced by smaller coefficients in the first two columns. Regarding social interaction, older adults living near parks exhibit insignificant coefficients, suggesting that social activities mitigate the adverse mental health effects of sedentary behavior. In contrast, individuals without park access show significant and larger coefficients, indicating that limited social interaction exacerbates mental health problems. In summary, these findings support Hypotheses 3a and 3b.

**Table 8:** Moderating effect—older adults' population.

	**Male-dominated caregivers**	**Female-dominated caregivers**	**Parks are nearby**	**No parks are nearby**
Sedentary	0.0042^*^	0.0029^**^	0.0010	0.0036^***^
	(0.0022)	(0.0012)	(0.0027)	(0.0012)
Covariates	Yes	Yes	Yes	Yes
Fixed-effects	Yes	Yes	Yes	Yes
Constant	22.9020^***^	17.8048^***^	23.4688^***^	21.0227^***^
	(3.9774)	(2.4768)	(6.6071)	(2.3328)
Obs.	1,629	5,484	1,334	5,540
R^2^	0.949	0.936	0.949	0.938

Significance: ^*^, ^**^, and ^***^ denote significance at 10%, 5%, and 1% level, respectively.

The dependent variable is Mental health 1.

All models include fixed effects and all covariates.

### Mediating effect

The literature suggests that obesity is a potential channel underlying our results. However, it remains necessary to test whether this channel is valid in our context. To address this, we use BMI, overweight status, and waist circumference as mediating factors. In the CHNS, weight is measured to the nearest 0.1 kg using a calibrated beam scale with participants wearing lightweight clothing, while height is measured to the nearest 0.1 cm using a portable stadiometer with participants barefoot (90). BMI (kg/m^2^) is calculated as a mediating factor, and overweight status is defined as a BMI > 25 based on Chinese criteria (91). Waist circumference is measured at the midpoint between the bottom of the rib cage and the top of the iliac crest at the end of exhalation. Table 9 presents the empirical results. The findings show that increased leisure sedentary time is associated with higher BMI, a greater likelihood of being overweight, and higher waist circumference. These results support Hypothesis 4.

**Table 9:** Mediating effect—BMI and obesity.

	**BMI**	**Overweight**	**Waist circumference**
Sedentary	0.0004^**^	0.0001^*^	0.0016^*^
	(0.0002)	(0.0000)	(0.0009)
Covariates	Yes	Yes	Yes
Fixed-effects	Yes	Yes	Yes
Constant	22.8958^***^	0.2866^***^	87.0944^***^
	(0.3567)	(0.0675)	(1.7050)
Obs.	23,693	23,693	23,693
R^2^	0.918	0.848	0.826

Significance: ^*^, ^**^, and ^***^ denote significance at 10%, 5%, and 1% level, respectively.

The dependent variable is Mental health 1.

All models include fixed effects and all covariates.

### Heterogeneity analyses

In our analysis, we control for occupational sedentary time to address concerns about omitted variable bias in the relationship between occupational sedentary time and mental health. To further examine the role of occupational sedentary time, we conduct a heterogeneous analysis by occupational type. The sample is divided into three categories: light physical work, medium physical work, and heavy physical work. Table 10 presents the results. Individuals engaged in light and medium physical work show a higher risk of mental health issues compared to those in heavy physical work. Moreover, the coefficients in the first two columns are larger than the baseline results, suggesting that individuals with high levels of both leisure and occupational sedentary time face worse mental health outcomes.

**Table 10:** Heterogeneity—occupational types.

	**Light physical work**	**Medium physical work**	**Heavy physical work**
Sedentary	0.0022^*^	0.0023^*^	0.0018
	(0.0013)	(0.0013)	(0.0012)
Covariates	Yes	Yes	Yes
Fixed-effects	Yes	Yes	Yes
Constant	26.6443 ^***^	26.9899^***^	27.2055^***^
	(2.8826)	(2.9286)	(2.7974)
Obs.	10,324	10,160	10,450
R^2^	0.972	0.972	0.971

Significance: ^*^, ^**^, and ^***^ denote significance at 10%, 5%, and 1% level, respectively.

The dependent variable is Mental health 1.

All models include fixed effects and all covariates.

### Robustness analyses

Our analysis focuses on adults who have completed formal education as the main sample. To test whether this sample restriction affects the generalizability of the findings, we perform several subsample analyses to ensure robustness. First, we exclude groups that may bias the results, including the oldest-old population (aged ≥80 years), individuals reporting poor health status, those diagnosed with genetic diseases, recently retired individuals, and individuals diagnosed with muscle diseases. The first three groups are more likely to experience mental health issues, while the last two are more likely to engage in sedentary behavior. Additionally, we randomly select 80% of the sample to further test the model's robustness. Tables 11, 12 present the robustness checks. The results across all subsamples remain consistent with the baseline findings, confirming the reliability of the empirical results.

**Table 11:** Robustness checks 1.

	**Excluding the oldest-old population**	**Individuals in good physical health**	**Individuals without genetic diseases**
Sedentary	0.0016^**^	0.0014^**^	0.0018^***^
	(0.0007)	(0.0007)	(0.0007)
Covariates	Yes	Yes	Yes
Fixed-effects	Yes	Yes	Yes
Constant	25.4739^***^	23.9203^***^	25.4764^***^
	(1.4601)	(1.4557)	(1.4364)
Obs.	23,094	22,463	23,498
R^2^	0.960	0.961	0.960

Significance: ^*^, ^**^, and ^***^ denote significance at 10%, 5%, and 1% level, respectively.

The dependent variable is Mental health 1.

All models include fixed effects and all covariates.

**Table 12:** Robustness checks 2.

	**80% samples**	**Excluding recently retired individuals**	**Individuals without muscular diseases**
Sedentary	0.0024^***^	0.0016^**^	0.0014^**^
	(0.0008)	(0.0007)	(0.0007)
Covariates	Yes	Yes	Yes
Fixed-effects	Yes	Yes	Yes
Constant	24.5096^***^	25.4739^***^	23.9203^***^
	(1.7356)	(1.4601)	(1.4557)
Obs.	18,924	23,094	22,463
R^2^	0.965	0.960	0.961

Significance: ^*^, ^**^, and ^***^ denote significance at 10%, 5%, and 1% level, respectively.

The dependent variable is Mental health 1.

All models include fixed effects and all covariates.

## Discussion

In recent decades, technological advancements have transformed daily life, offering convenience and comfort while increasing sedentary time. Prolonged sedentary time has become a defining feature of modern life and a pressing public health concern. Simultaneously, the fast-paced nature of contemporary life has heightened stress levels from work and personal responsibilities, leading many individuals to experience psychological sub-health (92). Thus, understanding the relationship between sedentary time and mental health is therefore crucial.

This article investigates the relationship between leisure sedentary time and mental health issues using a representative longitudinal sample of the Chinese population. By employing Ordinary Least Squares (OLS) regression, instrumental variable methods, and the Difference-in-Differences framework, we find that prolonged leisure sedentary time negatively affects mental health, primarily through its impact on obesity. Our findings align with existing literature on the adverse effects of sedentary behavior on mental health, which has primarily relied on cross-sectional data.

We also identify several moderating factors unique to the Chinese context that may mitigate the negative effects of sedentary time. These include promoting health literacy and reducing sedentary time across the population, as well as fostering caregiving by women and enhancing social interactions for older adults. First, in January 2008, the Chinese Ministry of Health issued the “Chinese Resident Health Literacy—Basic Knowledge and Skills (Trial),” the first government document defining health literacy. This initiative marked a critical step in promoting health education in China. However, subsequent surveys revealed that health literacy rates were only 8.8% in 2012 and 11.58% in 2016 (93, 94). By 2023, the rate had improved to 29.7% (95), but it remains low compared to other countries. Improving health literacy is therefore essential to preventing health issues and promoting wellbeing. Second, the eldercare system in China predominantly follows the “9073” structure: 90% of older adults are cared for by family members, 7% by community services, and 3% in nursing homes. Filial piety, a cultural virtue emphasizing respect and care for one's parents, underpins this model. Traditionally, sons bore the primary responsibility for eldercare. However, with the One-Child Policy and rising female workforce participation, daughters now provide both practical and emotional support (96). Intergenerational support, particularly emotional support, has been shown to reduce the stress of aging and positively influence health outcomes. Third, social interactions, which include personal and environmental interactions, are vital for mental health and psychological wellbeing. Older adults who actively engage in social activities report lower levels of loneliness (97). Accessible destinations such as parks, squares, and cultural facilities foster social connections. Parks, in particular, have been shown to encourage physical activity and social ties, contributing to better mental health and reduced sedentary behaviors (98, 99).

Regarding mechanisms, we identify obesity as a primary mediating factor. Additionally, prior research suggests other pathways: screen-based activities may overstimulate the central nervous system, increasing anxiety (100), while disrupted sleep patterns linked to screen use can exacerbate anxiety (101). Prolonged sedentary behavior is also associated with increased inflammatory markers, which may impact mental health (102).

Our findings yield three policy implications. First, prolonged sedentary time worsens mental health, underscoring the need for government campaigns to raise awareness about its risks. Efforts should focus on improving health literacy, as health literacy interventions have been shown to foster behavior change (103). For example, the Healthy China 2030 Plan identifies health literacy as a key strategy for improving public health. Additionally, local governments should also provide accessible fitness equipment and promote physical activity through initiatives like urban greenways, which have been shown to reduce sedentary behavior (104). Second, governments should support female caregivers, especially daughters, who play a crucial role in eldercare. Policies could include financial incentives or tax benefits for caregiving daughters and encourage employers to offer flexible work arrangements for women engaged in eldercare, such as remote work or part-time schedules. Third, creating accessible and engaging spaces for older adults can reduce sedentary behavior. Governments could subsidize social activities, such as senior tour groups, while communities could promote participation in local events to foster social interaction.

This study also has limitations. First, the findings are rooted in China's cultural and institutional context. Factors such as Confucian traditions and the health system shape health behaviors and outcomes, so caution is needed when generalizing these results to other countries. Replication studies in diverse settings could enhance external validity. Second, sedentary time was self-reported, which may lead to underestimation. Combining self-reports with accelerometer data in future research could improve accuracy. Third, while the fixed-effects method, IV method and DID method mitigate endogeneity, residual bias or confounding variables may persist. Experimental designs could further validate these findings. Finally, data limitations prevented an in-depth exploration of biological pathways, which future studies could address.

## Conclusion

In conclusion, our research finds that prolonged leisure sedentary time negatively affects mental health. Additionally, our study reveals moderating roles of promoting health literacy and reducing sedentary time across the population, as well as fostering caregiving by women and enhancing social interactions for older adults. Finally, we identify obesity as a primary mediating factor. The above findings are significant given the global rise in both sedentary behaviors and mental health challenges.

## Data Availability

The original contributions presented in the study are included in the article/supplementary material, further inquiries can be directed to the corresponding author.

## Footnotes

^1^The dietary knowledge index includes 1) Eating vegetables and fruits is beneficial to health; 2) Eating less sugar is beneficial to health; 3) Eating a variety of foods is beneficial to health; 4) Eating less high-fat food is beneficial to health; 5) Eating dairy products is beneficial to health; 6) Eating soy products is beneficial to health.
